# Supplementary material for: Designing isolation guidelines for COVID-19 patients with rapid antigen tests
Source: Nat Commun. 2022 Aug 20;13:4910. doi: 10.1038/s41467-022-32663-9 (PMC9392070; doi:10.1038/s41467-022-32663-9)
Supplement: Supplementary file 1 — Supplementary Information [file 41467_2022_32663_MOESM1_ESM.pdf]

Supplementary Information

Designing isolation guidelines for COVID-19 patients with rapid antigen tests

Yong Dam Jeong[1,2,†], Keisuke Ejima[3,4,†,*], Kwang Su Kim[1,5†], Woo Joohyeon[1], Shoya Iwanami[1], Yasuhisa Fujita[1], Il Hyo Jung[2], Kazuyuki Aihara[6], Kenji Shibuya[4], Shingo Iwami[1, 7,8,9,10,11*], Ana I. Bento[3‡] and Marco Ajelli[12‡]

[1]interdisciplinary Biology Laboratory (iBLab), Division of Biological Science, Graduate School of Science, Nagoya University, Nagoya, Japan. [2]Department of Mathematics, Pusan National University, Busan, South Korea. [3]Department of Epidemiology and Biostatistics, Indiana University School of Public Health-Bloomington, IN, USA. [4]The Tokyo Foundation for Policy Research, Tokyo, Japan. [5]Department of Scientific computing, Pukyong National University, Busan, South Korea. [6]International Research Center for Neurointelligence, The University of Tokyo, Tokyo, Japan. [7]Institute of Mathematics for Industry, Kyushu University, Fukuoka, Japan. [8]Institute for the Advanced Study of Human Biology (ASHBi), Kyoto University, Kyoto, Japan. [9]NEXT-Ganken Program, Japanese Foundation for Cancer Research (JFCR), Tokyo, Japan. [10]Interdisciplinary Theoretical and Mathematical Sciences Program (iTHEMS), RIKEN, Saitama, Japan. [11]Science Groove Inc., Fukuoka, Japan. [12]Laboratory for Computational Epidemiology and Public Health, Department of Epidemiology and Biostatistics, Indiana University School of Public Health-Bloomington, IN, USA

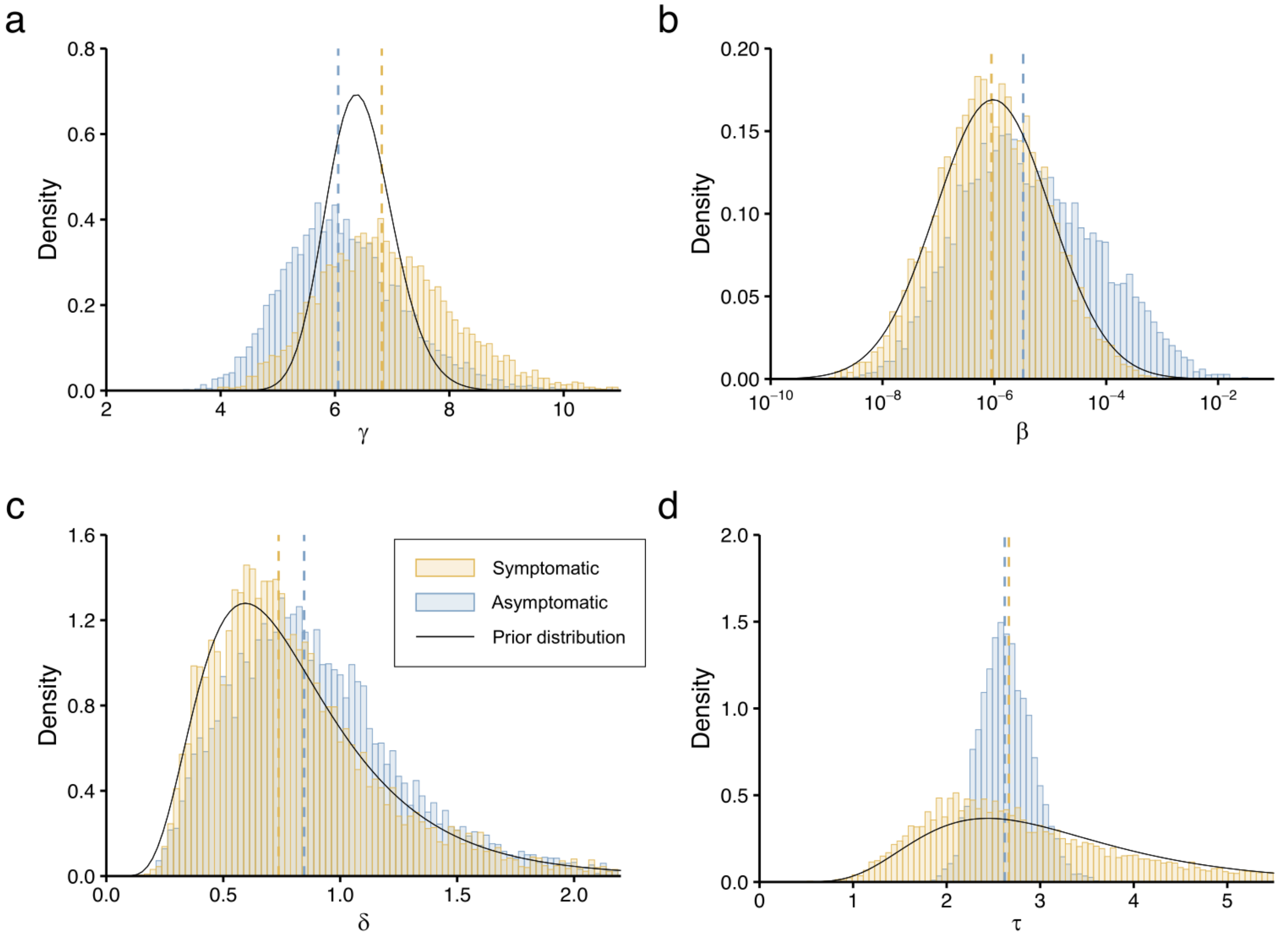

Supplementary Figure 1. Posterior distributions of the model parameters for symptomatic and asymptomatic patients. a. Maximum rate constant for viral replication (γ), **b.** Rate constant for virus infection (β), **c.** Death rate of infected cells (δ), and **d.** Interval between infection to symptom onset or diagnosis (τ). In each panel, the black solid line represents the prior distribution of the model parameter. The dashed vertical lines indicate the median values. Each histogram is based on 10,000 samples from the posterior distribution.

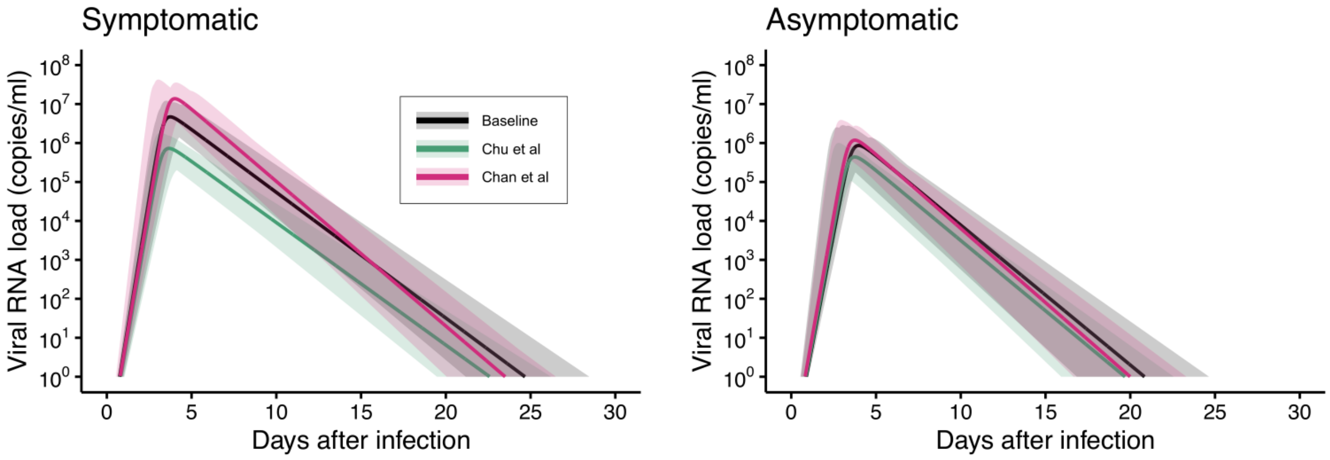

Supplementary Figure 2. Estimated viral load curves for symptomatic and asymptomatic patients with different conversion equations. The solid lines are the estimated viral load curves for the best fit parameters of fixed effect using different conversion equations. The shaded regions correspond to 95% predictive intervals. Viral load was calculated from cycle threshold values using the conversion formulas: $\log_{10}(\text{Viral load [copies/mL]}) = -0.29 \times \text{Ct values [cycles]} + 11.58$ (Chu et al) and $\log_{10}(\text{Viral load [copies/mL]}) = -0.40 \times \text{Ct values [cycles]} + 17.03$ (Chan et al).

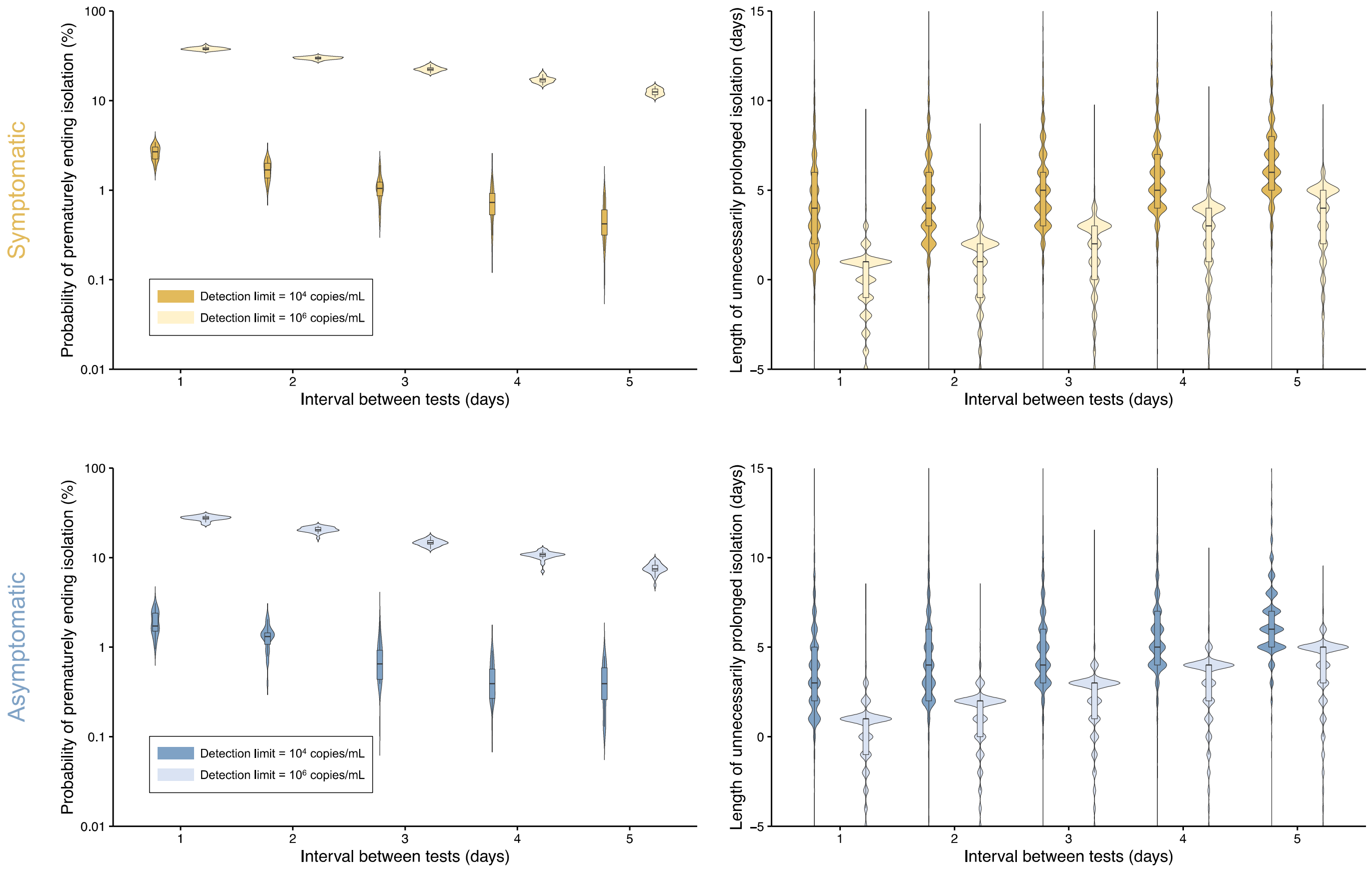

Supplementary Figure 3. Distributions of the risk and the burden with representative scenarios. Distributions of probability of prematurely ending isolation (risk) and length of unnecessarily prolonged isolation (burden) with representative scenarios (infectiousness threshold: $10^{5.0}$ copies/mL, detection limit: $10^{4.0}$ copies/mL [corresponding to Figure 2] and $10^{6.0}$ copies/mL [corresponding to Figure 3]; two consecutive negative results) varying the interval between tests (1 to 5 days). The violin plots show the kernel probability density, whereas the box plots show the median (50 percentile; bold lines) and interquartile ranges (25 and 75 percentiles; boxes). Note that the computed values are based on 100 simulations with 1,000 patients each for symptomatic and asymptomatic cases, respectively.

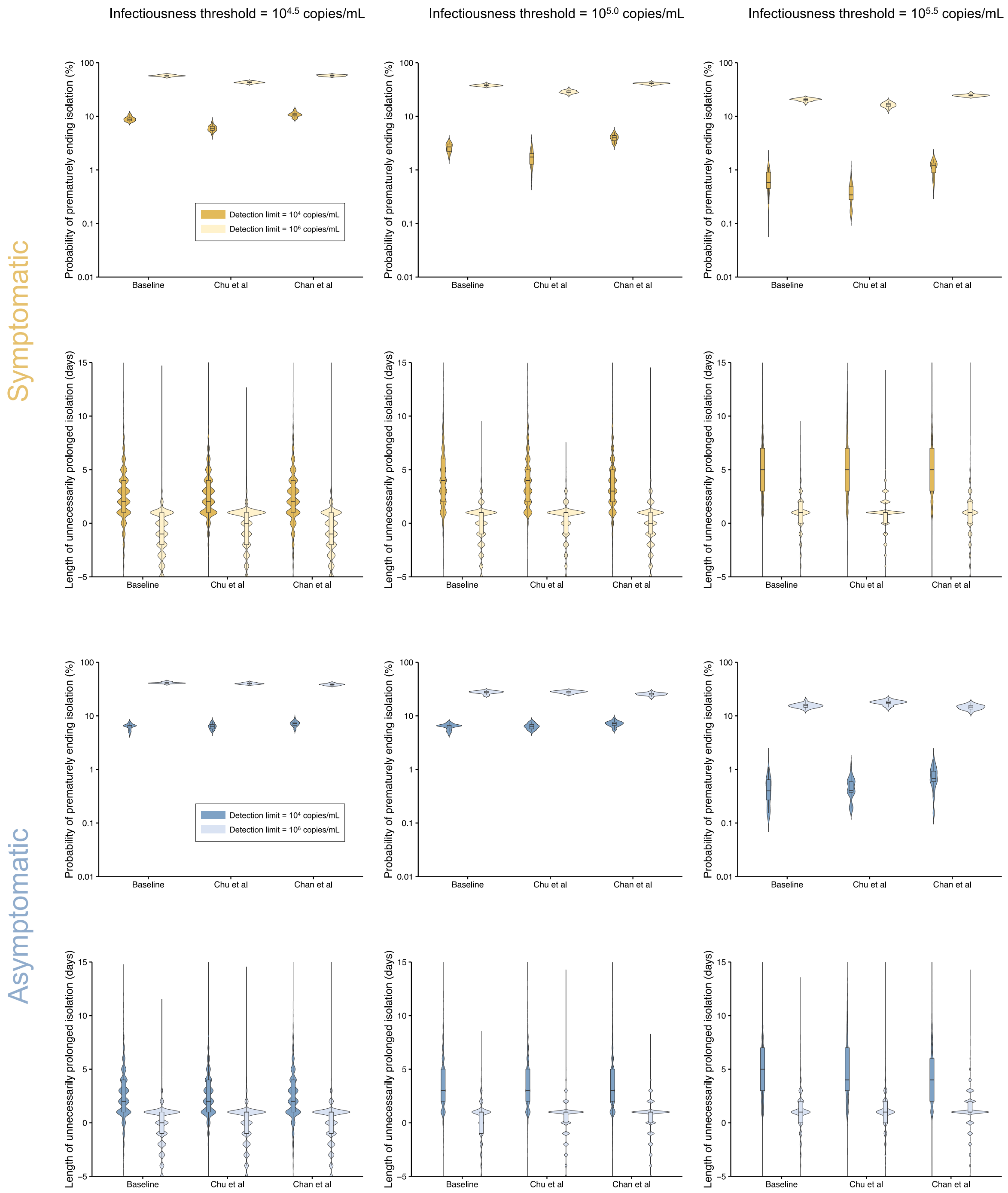

Supplementary Figure 4. Comparison of risk and cost with a representative scenario with different conversion equations. Distributions of probability of prematurely ending isolation (risk) and length of unnecessarily prolonged isolation (burden) with a representative scenario (consecutive negative results: twice, interval of tests: 1 day). The violin plots show the kernel probability density, whereas the box plots show the median (50 percentile; bold lines) and interquartile ranges (25 and 75 percentiles; boxes). Note that the computed values are based on 100 simulations with 1,000 patients each for symptomatic and asymptomatic cases, respectively.

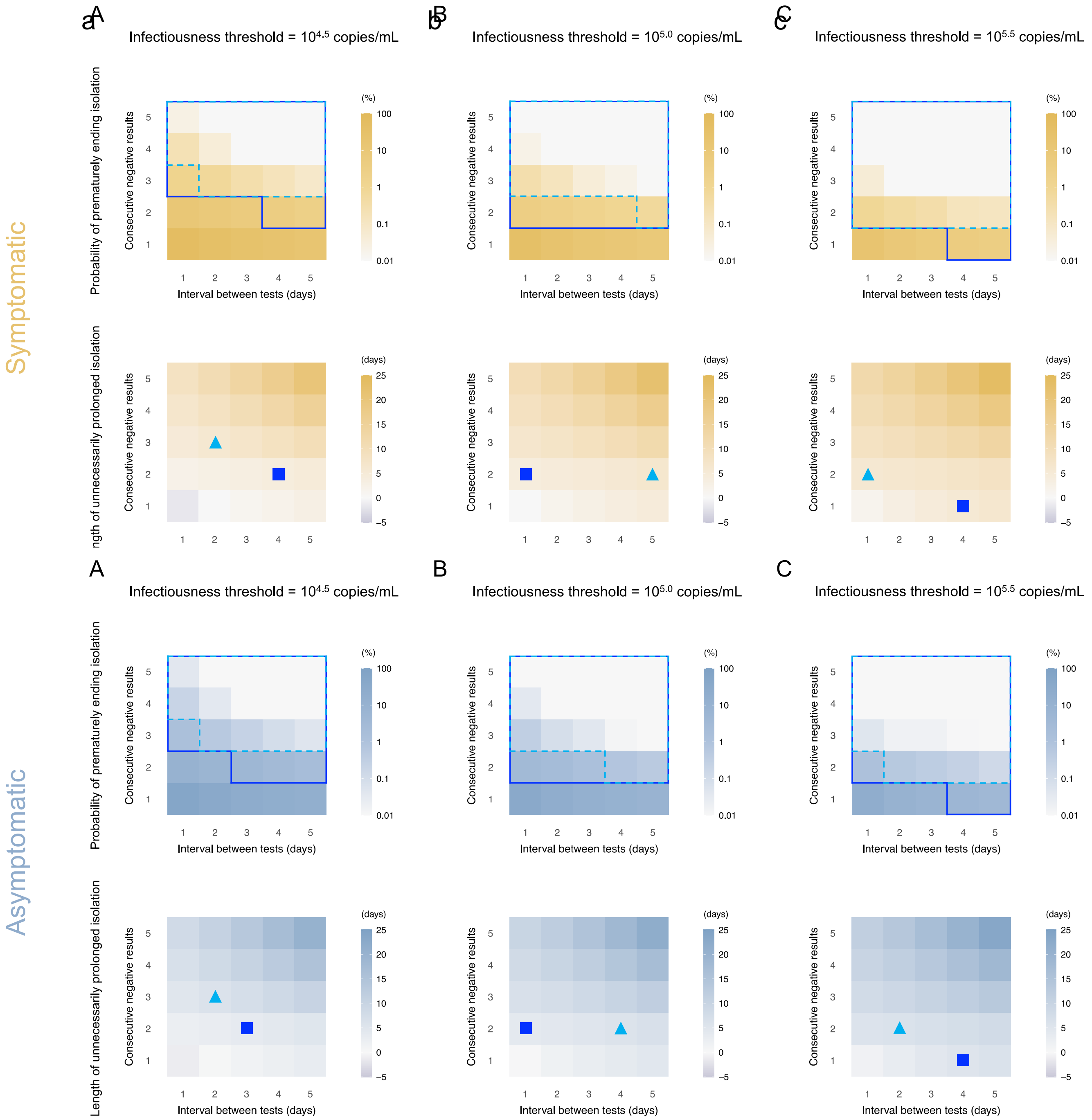

Supplementary Figure 5. Optimal isolation guideline for symptomatic and asymptomatic cases with different guidelines assuming the isolation and the first test were performed 5 days after infection. Detection limit was fixed at $10^{4.0}$ copies/mL. Infectiousness threshold value were varied: **a.** $10^{4.5}$ copies/mL, **b.** $10^{5.0}$ copies/mL, and **c.** $10^{5.5}$ copies/mL. Note that the computed values are based on 100 simulations with 1,000 patients each for symptomatic and asymptomatic cases, respectively.

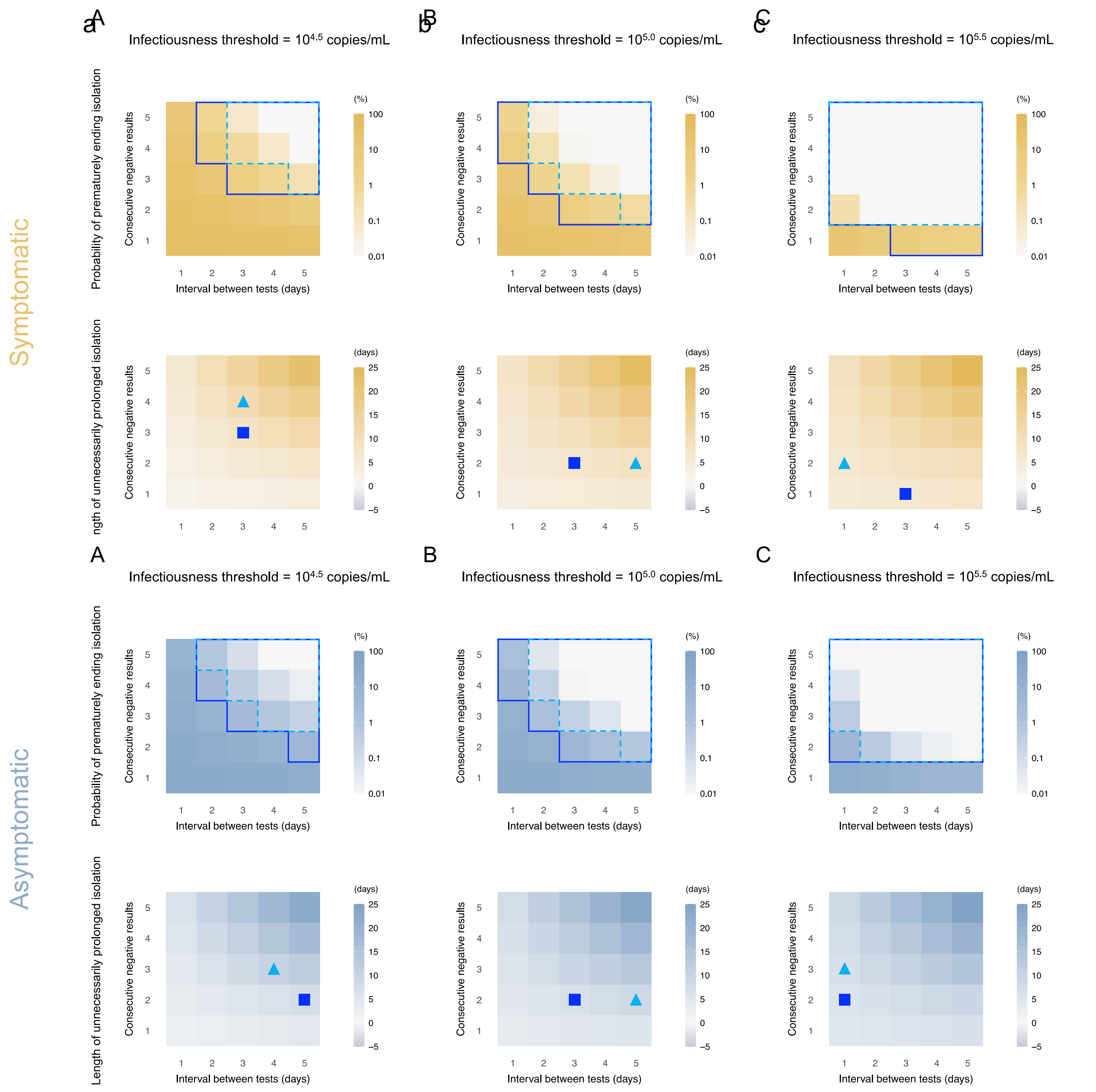

Supplementary Figure 6. Optimal isolation guideline for symptomatic and asymptomatic cases with different guidelines assuming the error is fixed over time for each patient. Detection limit was fixed at $10^{4.0}$ copies/mL. Infectiousness threshold value were varied: **a.** $10^{4.5}$ copies/mL, **b.** $10^{5.0}$ copies/mL, and **c.** $10^{5.5}$ copies/mL. Note that the computed values are based on 100 simulations with 1,000 patients each for symptomatic and asymptomatic cases, respectively.

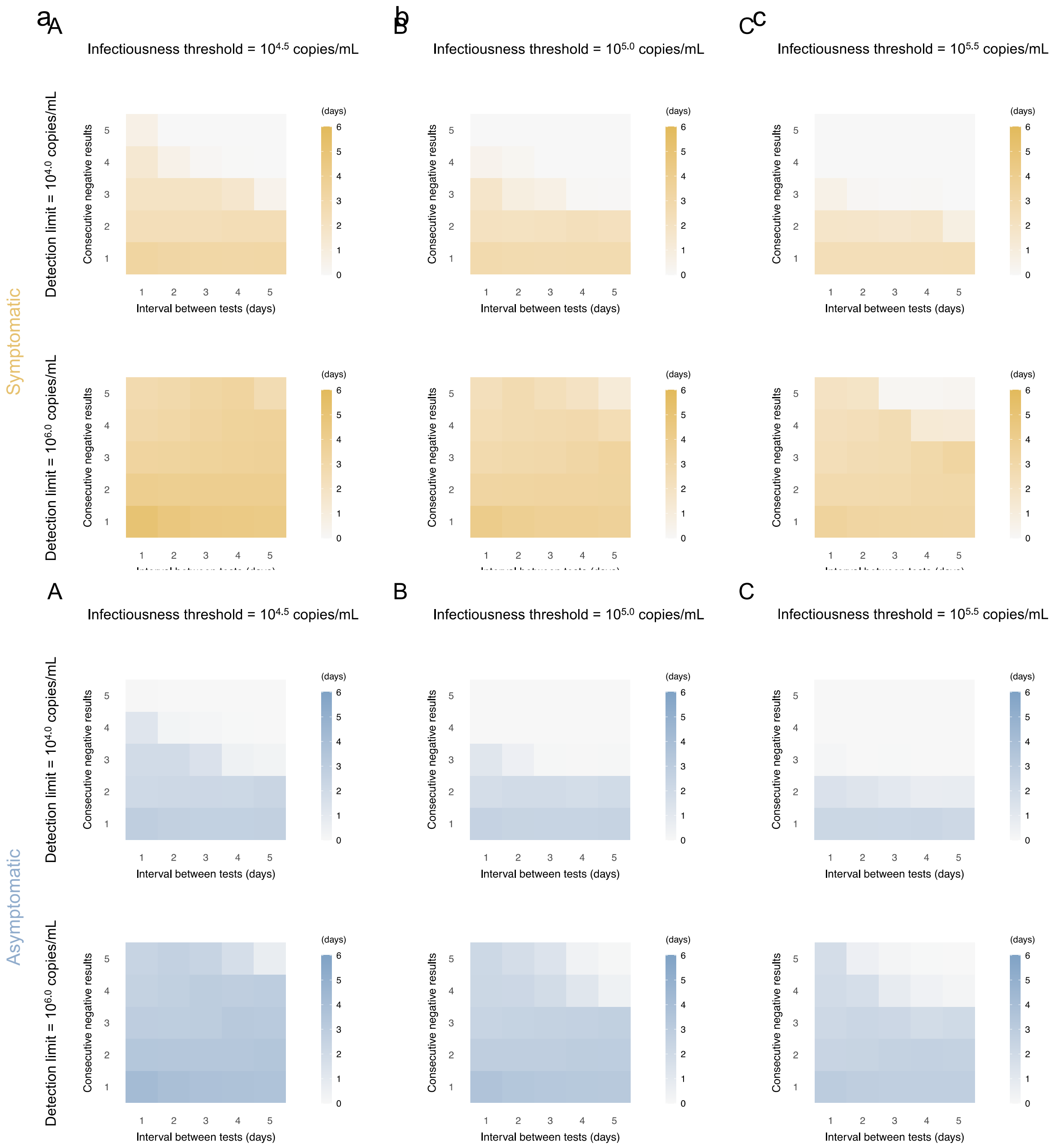

Supplementary Figure 7. Mean number of days remain infectious after isolation among infectious patients with different guidelines. Infectiousness threshold value were varied: **a.** $10^{4.5}$ copies/mL, **b.** $10^{5.0}$ copies/mL, and **c.** $10^{5.5}$ copies/mL. Detection limit was varied: $10^{4.0}$ copies/mL and $10^{6.0}$ copies/mL. Note that the computed values are based on 100 simulations with 1,000 patients each for symptomatic and asymptomatic cases, respectively.

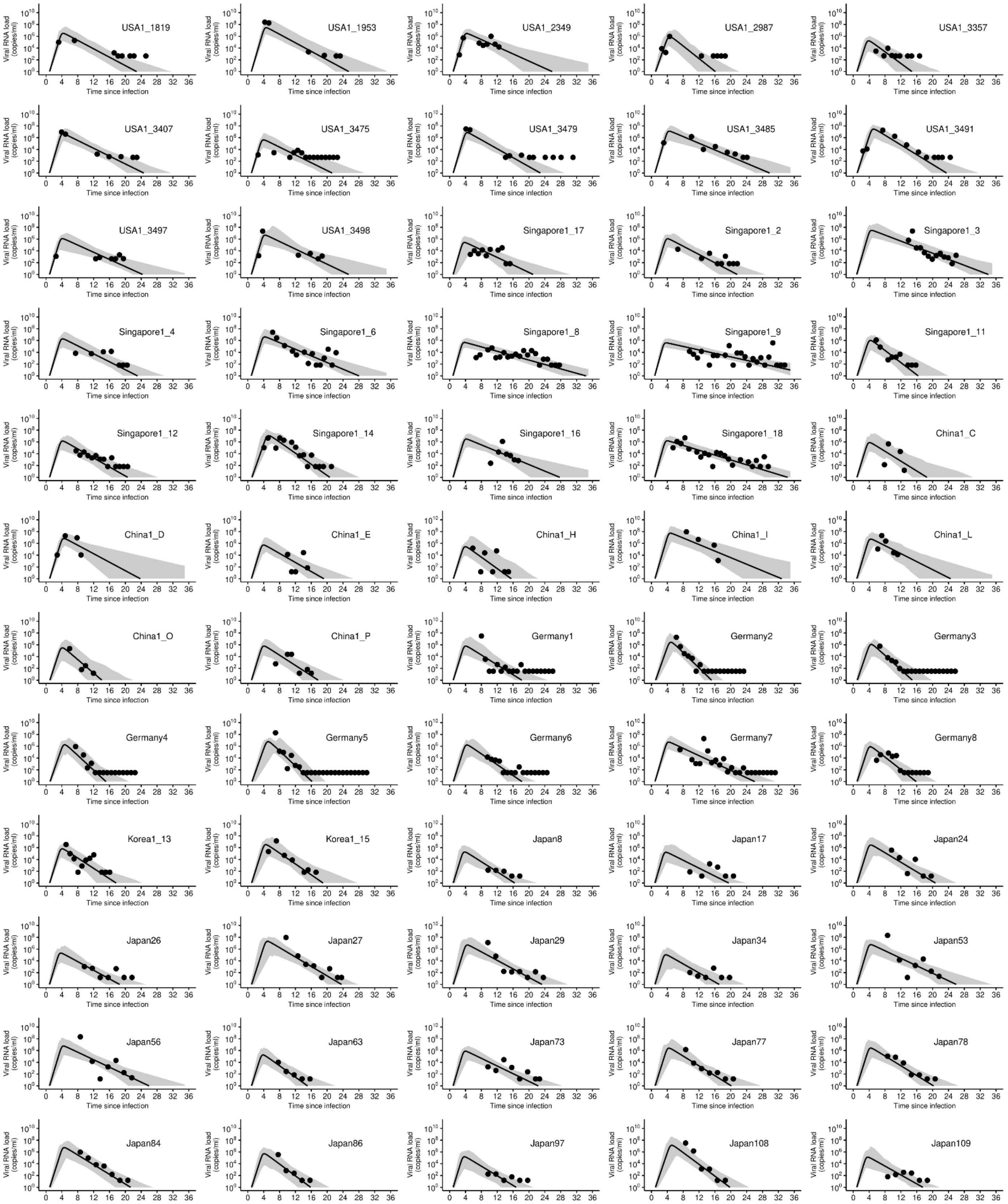

Supplementary Figure 8. Viral load dynamics for each patient. Measured viral load (open circles) and the best-fit curves (with the best fit parameters; solid lines) are shown. Note that, as we analyzed 210 patients in total, we randomly selected 60 symptomatic and asymptomatic individuals from different locations to be included in the figure. The shaded regions correspond to 95% predictive intervals.

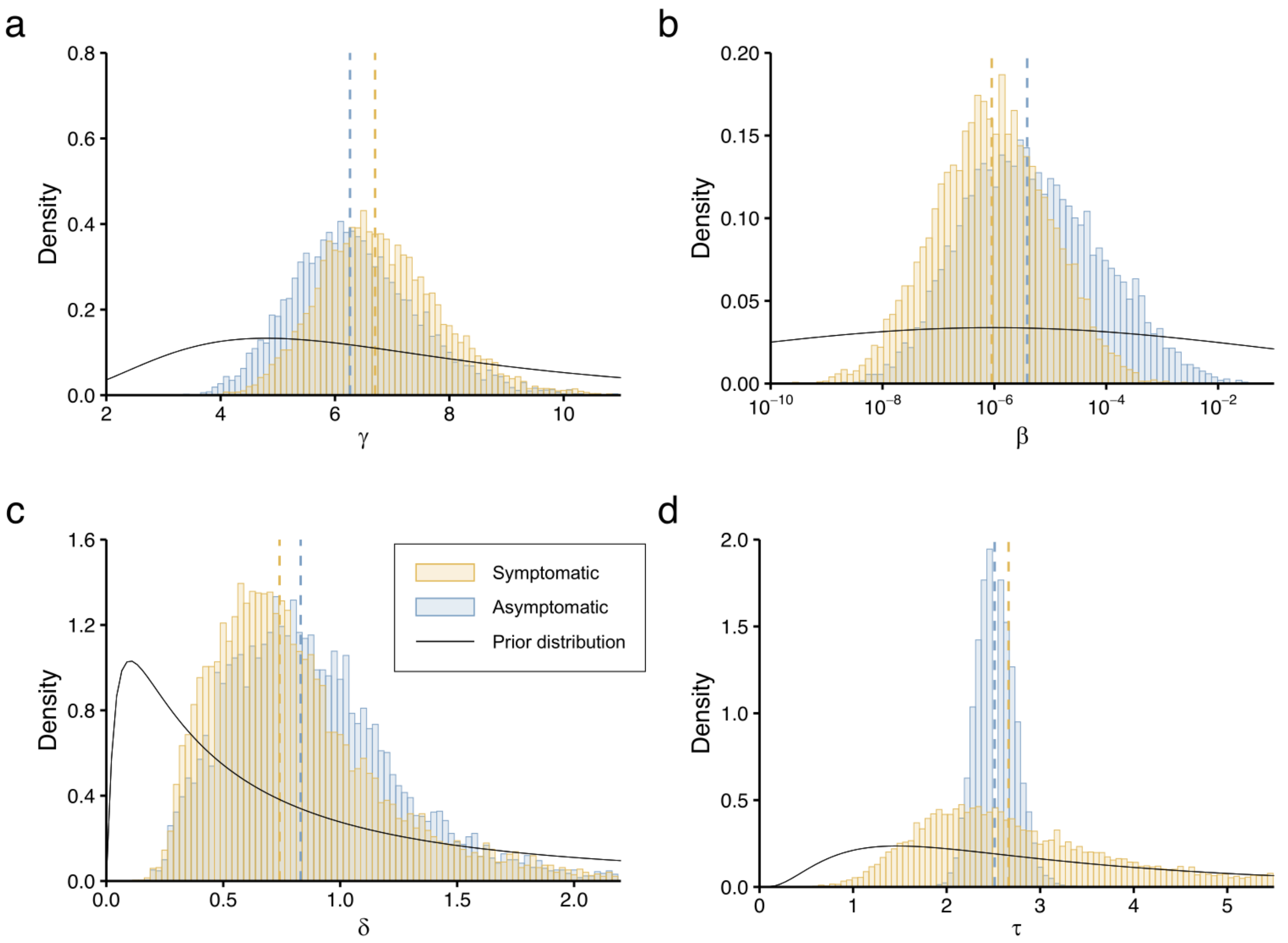

Supplementary Figure 9. Posterior distributions of the model parameters for symptomatic and asymptomatic patients. a. Maximum rate constant for viral replication (γ), **b.** Rate constant for virus infection (β), **c.** Death rate of infected cells (δ), and **d.** Interval between infection to symptom onset or diagnosis (τ). In each panel, the black solid line represents the prior distribution of the model parameter. The dashed vertical lines indicate the median values. We set the prior distributions of the parameters γ, β, δ, and τ as $lognorm(1.86{,}0.54)$, $lognorm(-13.87{,}11.8)$, $lognorm(-0.30{,}1.41)$, and $lognorm(1.06{,}0.82)$, respectively (note they are the same for both symptomatic and asymptomatic patients) Each histogram is based on 10,000 samples from the posterior distribution. Note that, as compared with Supplementary Figure 1, different prior distributions are used here.

Supplementary Table 1. Medians and 95% CIs of model fixed effect parameters (in parentheses) computed from the posterior distributions, the variance of random effect, and the variance of the error in viral load

Parameters	Symbol	Unit	Symptomatic		Asymptomatic	
			Median of fixed effect (95% CI)	Variance of random effect	Median of fixed effect (95% CI)	Variance of random effect
Maximum rate constant for viral replication	γ	day^{-1}	6.81 (4.88 to 9.49)	0.029	6.06 (4.37 to 8.42)	0.028
Rate constant for virus infection	β	$(copies/mL)^{-1}$ day^{-1}	9.41×10^{-7} (1.24×10^{-8} to 7.14×10^{-5})	4.882	4.15×10^{-6} (1.65×10^{-8} to 1.04×10^{-3})	7.952
Death rate of infected cells	δ	day^{-1}	0.74 (0.31 to 1.79)	0.202	0.83 (0.36 to 1.94)	0.187
Interval between infection to symptom onset (for symptomatic cases) or diagnosis (for asymptomatic cases)	τ	day	2.8 (1.2 to 6.3)	0.182	2.6 (2.1 to 3.2)	0.011
Variance of the error	σ^2	$log(copies/mL)^2$	1.64		1.82	

Supplementary Table 2. Comparison of the posterior distributions with different prior distributions. Medians and 95% CIs of model fixed effect parameters (in parentheses) computed from the posterior distributions.

	Symptomatic		Asymptomatic	
	**Informative prior distributions (Suppl. Fig. 1)**	**Uninformative prior distributions (Suppl. Fig. 9)**	**Informative prior distributions (Suppl. Fig. 1)**	**Uninformative prior distributions (Suppl. Fig. 9)**
γ	6.81<br>(4.88 to 9.49)	6.69<br>(4.96 to 9.11)	6.06<br>(4.37 to 8.42)	6.27<br>(4.45 to 8.82)
β	9.41×10^{-7}<br>(1.24×10^{-8} to 7.14×10^{-5})	8.77×10^{-7}<br>(9.96×10^{-9} to 7.96×10^{-5})	4.15×10^{-6}<br>(1.65×10^{-8} to 1.04×10^{-3})	5.10×10^{-6}<br>(3.46×10^{-8} to 1.30×10^{-3})
δ	0.74<br>(0.31 to 1.79)	0.75<br>(0.32 to 1.86)	0.83<br>(0.36 to 1.94)	0.80<br>(0.33 to 1.81)
τ	2.8<br>(1.2 to 6.3)	2.8<br>(1.2 to 7.5)	2.6<br>(2.1 to 3.2)	2.5<br>(2.2 to 2.9)

Supplementary Note 1: Parameter estimation

Model fitting procedure

Nonlinear mixed effects model was employed to fit the viral dynamics model to the viral load data using MONOLIX 2019R2 (www.lixoft.com). The nonlinear mixed-effect model considers fixed effect (the same among individuals. i.e., population parameter) and random effect (different between individuals) for each parameter. Specifically, the parameter for each patient k (i.e., individual parameter), $\theta_k (= \theta \times e^{\eta_k})$ is a product of θ (fixed effect) and η_k (random effect). The random effect is assumed to follow the normal distribution: $N(0, \Omega)$. Note that the random effect captures individual variability. The population parameter θ (i.e., fixed effect) were estimated using Stochastic Approximation Expectation Maximization (SAEM). Individual parameters θ_k (i.e., fixed + random effect) are subsequently estimated using Markov Chain Monte Carlo (MCMC)[1,2]. The SAEM and MCMC algorisms were employed because the likelihood functions were complicated and high-dimensional parameter space was needed to be explored. The parameter estimation process is composed of two phases: exploring phase and smoothing phase. The parameters space is explored to find the region of maximum likelihood (objective function) in the first phase and the identified parameter region is smoothed in the second phase. A detailed procedure is described as follows:

Population parameter (i.e., fixed effect parameter)

1) Exploring phase

(Step 1) Set the prior distributions of the population parameters. We set the prior distributions of the parameters γ, β, δ, and τ as $lognorm(1.86, 0.09)$, $lognorm(-13.87, 2.36)$, $lognorm(-0.30, 0.47)$, and $lognorm(1.06, 0.41)$, respectively (note they are the same for both symptomatic and asymptomatic patients). To guarantee the positiveness of the parameters (i.e., negative values do not biologically make sense), lognormal distributions were used as prior distributions. These prior distributions correspond to the posterior distributions obtained in a previous study [3]. To assess the reliability of our results, we also performed a sensitivity analysis where we used uninformative prior distributions. As shown in **Supplementary**

Figure 9 and **Supplementary Table 2**, the obtained results are robust to changes of the prior distributions.

(Step 2) Using MCMC, generate the conditional distribution $p(\theta_k | y_k, \theta^i)$, where θ_k and y_k are a set of individual parameters (i.e., γ, β, δ, and τ) and observed viral load data of patient k ($k = 1,2,\cdots,N$), and θ^i is a set of population parameters at iteration i.

(Step 3) Sample individual parameters θ_k^i from the conditional distribution $p(\theta_k | y_k, \theta^i)$ generated in Step 2.

(Step 4) Calculate the new population parameters from the individual parameters θ_k^i for $k = 1,2,\cdots,N$:

$$\theta^{i+1} = \frac{1}{N}\sum_{k=1}^{N} \theta_k^i.$$

(Step 5) For each iteration i (repeating Steps 2-4), check the convergence indicator, which is defined as the joint probability distribution of the observations and individual parameters: $p(y_k, \theta_k^i; \theta^i)$. $p(y_k, \theta_k^i; \theta^i)$ is derived using Bayes' theorem: $p(y_k | \theta_k^i; \theta^i)\ p(\theta_k^i; \theta^i)$. Note that $p(y_k | \theta_k^i; \theta^i)$ and $p(\theta_k^i | \theta^i)$ are assumed to follow a normal distribution and a lognormal distribution, respectively. The convergence indicator aggregates the information from all parameters and detects if the SAEM algorithm has already converged or not. Also, it serves as the auto-stop criteria to switch from the exploring phase to the smoothing phase [4].

The estimated population parameters converge to a neighborhood region of the maximum likelihood estimate. Note that the maximum number of iterations in the exploring phase was set as 5,000.

2) Smoothing phase

(Step 1) Repeat Steps 2-3 of *Exploring phase*.

(Step 2) Calculate the new population parameters from the individual parameters of all previous smoothing iterations:

$$\theta^{i+1} = \frac{1}{i}\left[\frac{1}{N}\sum_{k=1}^{N} \theta_k^1 + \frac{1}{N}\sum_{k=1}^{N} \theta_k^2 + \cdots + \frac{1}{N}\sum_{k=1}^{N} \theta_k^i\right] = \theta^i + \frac{1}{i}\left[\frac{1}{N}\sum_{k=1}^{N} \theta_k^i - \theta^i\right].$$

The estimated population parameters converge to the maximum likelihood estimate by accumulating the information of all previous iterations. Note that the maximum number of iterations in the smoothing phase was set as 200.

Individual parameter (i.e., fixed + random effect parameters)

Individual parameters were estimated as empirical bayes estimates (EBEs). The EBE is the most probable value of the individual parameters, which is the mode of the conditional distribution: $\hat{\theta}_k = \arg\max_{\theta_k} p(\theta_k | y_k, \hat{\theta})$, where $\hat{\theta}$ is the estimated population parameter.

Estimation of the variance of the error

We defined "error" the difference between the observed viral load and the viral load estimated by the model.

To estimate the variance of the error, we considered the following model:

$$y_{kj} = V(t_{kj}, \hat{\theta}_k) + \varepsilon_{kj}, \qquad 1 \le k \le N, \qquad 1 \le j \le n_k,$$

where y_{kj} and $V(t_{kj}, \hat{\theta}_k)$ are the observed and predicted viral load of patient k at time t_{kj} (at the jth observation). N is the total sample size and n_k is the number of observations for patient k. ε_{kj} is the residual error that is assumed to follow a normal distribution with mean 0 and variance σ^2. The variance of the error, σ^2, was thus estimated by fitting a normal distribution to the estimated residual errors: $\varepsilon_{kj} = y_{kj} - V(t_{kj}, \hat{\theta}_k)$.

Estimation of the time of infection

Because the time scales of the longitudinal viral load data are time after symptom onset (for symptomatic patients) or time after diagnosis (for asymptomatic patients), we shifted those time scale to the time since infection. For example, assuming that viral load for patient k is measured u_{kl} days after symptom onset ($l \in \{1,2, \dots n_k\}$, where n_k is the number of observation for patient k), the viral load is denoted as

$V(u_{kl} + \tau_k)$, where τ_k is the interval between infection to symptom onset for patient k (i.e., incubation period). τ_k is jointly estimated with the other parameter sets. To account for the left-censoring problem (i.e., when the viral load is under the detection limit, or tests negative), the likelihood function was developed using a left-truncated Gaussian distribution assuming such data are in the censoring interval (0 to the detection limit)[5].

1 *Conditional distribution*, <https://monolix.lixoft.com/tasks/conditional-distribution/> (2022).
2 *Population parameter estimation using SAEM*, <https://monolix.lixoft.com/tasks/population-parameter-estimation-using-saem/> (2022).
3 Jeong, Y. D. *et al.* Revisiting the guidelines for ending isolation for COVID-19 patients. *eLife* **10**, e69340, doi:10.7554/eLife.69340 (2021).
4 Traynard, P., Ayral, G., Twarogowska, M. & Chauvin, J. Efficient Pharmacokinetic Modeling Workflow With the MonolixSuite: A Case Study of Remifentanil. *CPT: Pharmacometrics & Systems Pharmacology* **9**, 198-210, doi:10.1002/psp4.12500 (2020).
5 Samson, A., Lavielle, M. & Mentré, F. Extension of the SAEM algorithm to left-censored data in nonlinear mixed-effects model: Application to HIV dynamics model. *Computational Statistics & Data Analysis* **51**, 1562-1574, doi:10.1016/j.csda.2006.05.007 (2006).
